# Why do Danish junior doctors choose general practice as their future specialty? Results of a mixed-methods survey

**DOI:** 10.1080/13814788.2019.1639668

**Published:** 2019-07-24

**Authors:** Gunver Lillevang, Mikael Henriksen, John Brodersen, Karolina Lewandowska, Niels Kristian Kjær

**Affiliations:** a Research Unit and Section of General Practice, Department of Public Health, Faculty of Health and Medical Sciences, University of Copenhagen, Copenhagen, Denmark;; b Copenhagen Academy for Medical Education and Simulation, Copenhagen, Denmark;; c Primary Healthcare Research Unit, University of Copenhagen, Copenhagen, Denmark;; d Ostend Medical Centre, Waiheke, New Zealand;; e Research Unit for General Practice, Institute of Public Health, University of Southern Denmark, Odense, Denmark

**Keywords:** Personnel selection, general practice, vocational guidance recruitment, specialty choice

## Abstract

**Background:** A well-staffed and an efficient primary healthcare sector is beneficial for a healthcare system but some countries experience problems in recruitment to general practice.

**Objectives:** This study explored factors influencing Danish junior doctors’ choice of general practice as their specialty.

**Methods:** This study is based on an online questionnaire collecting quantitative and qualitative data. Two focus-group interviews were conducted to inform the construction of the questionnaire to ensure high content validity. All Danish junior doctors participating in general practice specialist training in 2015 were invited to participate in the survey, from which both qualitative and quantitative data were collected. The data was analysed using systematic text condensation and descriptive statistics.

**Results:** Of 1099 invited, 670 (61%) junior doctors completed the questionnaire. Qualitative data: junior doctors found educational environments and a feasible work–life balance were important. They valued patient-centred healthcare, doctor–patient relationships based on continuity, and the possibility of organizing their work in smaller, manageable units. Quantitative data**:** 90.8% stated that the set-up of Danish specialist-training programme positively influenced their choice of general practice as their specialty. Junior doctors (80.4%) found that their university curriculum had too little emphasis on general practice, 64.5% agreed that early basic postgraduate training in general practice had a high impact on their choice of general practice as their specialty.

**Conclusion:** Several factors that might positively affect the choice of general practice were identified. These factors may hold the potential to guide recruitment strategies for general practice.

KEY MESSAGES

- Factors that can facilitate junior doctors’ choice of general practice.
- High-quality general practice training and early exposure to general practice, both undergraduate and postgraduate.
- Having influence on their working conditions, being independent, having good working conditions and a good work–life balance.
- A patient-centred approach with a good doctor–patient relationship.

## Introduction

It is established that a well-educated and well-organized primary healthcare sector is beneficial for the entire healthcare system [1,2]. Unfortunately, junior doctors’ attraction to general practice seems to be in decline and some countries experience problems with recruitment to general practice [3–7].

We know that a general practice-oriented undergraduate curriculum and early postgraduate exposure to general practice have a positive impact on recruitment to general practice [8–12]. We also know that senior doctor’s and society’s views on general practice affect junior doctors’ choice as does the perceived work–life balance [10,13,14].

Recruitment problems for general practice have reached Denmark [15], the setting for this study. Danish healthcare is organized in a national public system covering all citizens. GPs are private entrepreneurs governed through a collective agreement with the public contractors and GPs’ income is equivalent to hospital consultants’ [16]. General practice is the gatekeeper for the healthcare system [16].

**Figure 1.**
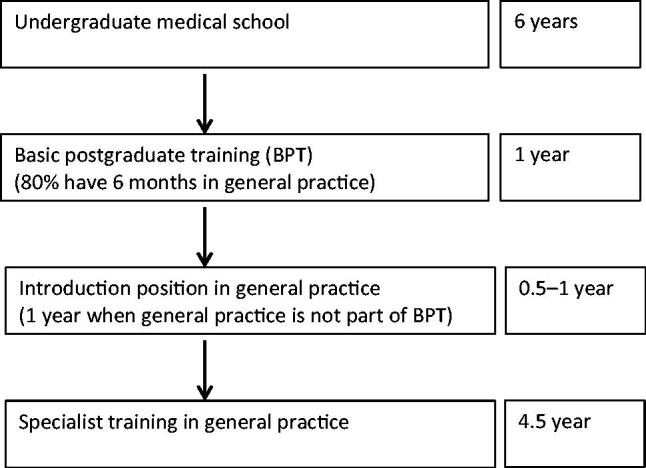
Flow diagram illustrating the educational career of Danish GPs.

The Danish specialist general practice training programme is five years, the same length as most other Danish hospital specialties [17]. A flow diagram illustrating the educational career of Danish GPs is shown in Figure 1. The inter-specialty competition is relatively low. Most specialties are relatively easily accessible for all graduate doctors. The selection process is based on defined entry criteria within each specialty.

General practice has been rated as one of the top five attractive specialties by Danish medical students [18]. Danish junior doctors tend to be older than their colleagues from countries with other educational set-ups due to later study start and longer maternity/paternity leave [19].

This study aimed to explore the motivations and factors influencing Danish junior doctors’ choice of general practice as a specialty to optimize recruitment to general practice.

## Methods

### Study design

A questionnaire survey collecting qualitative and quantitative data was conducted among Danish junior doctors in training positions for general practice.

### Ethics

The Danish National Committee in Health Research Ethics was consulted on the study design [20] and it was approved by each of the three medical postgraduate educational departments. The participants gave consent when entering the study. Data was anonymized prior to analysis.

### Construction of questionnaire

The questionnaire was constructed based on findings from focus groups and literature.

We conducted two focus-group interviews, inviting junior doctors training for general practice in two geographical areas (Zealand and Southern Denmark). Participants were recruited via social media and enrolled in the order they replied. Twelve junior doctors participated, ten females and two males, with six in each group, representing junior doctors at different stages of the training programme. The interviews focused on factors influencing the choice of general practice and on when this choice was made.

The interviews were transcribed. Two researchers independently analysed the text following a systematic text condensation approach establishing ‘meaning-carrying units’ and condensed these into generalized statements [21]. The analysis results were discussed with a third researcher. We applied a strict methodology, including researcher triangulation, and compared the degree of coherence between and within the two interviews following Malterud recommendations [22]. We only included topics discussed in both interviews.

The findings from the two groups were homogeneous. Seven generalised statements were identified in agreement by the researchers and were used to generate statements for the topics in the questionnaire (Box 1).

Box 1 The seven statements generated by the focus group data analysis.

1. The undergraduate curriculum matters in relation to the choice of general practice, since GP role models and the opportunity to work with patients in a GP setting boost interest in general practice.
2. The quality of the postgraduate GP specialist training programme influences the choice of general practice as specialty.
3. Junior doctors heading towards general practice appreciate working with a holistic and patient-centred approach.
4. Junior doctors chose GP specialist training based on an intention to settle as a GP in Denmark.
5. Junior doctors heading towards general practice appreciate that general practice is a smaller organization compared to the larger hospital departments.
6. The status as self-employed and responsible for management, and organization of work positively affects the choice of general practice.
7. Early postgraduate exposure to general practice positively influences the consideration of general practice as a career.

We performed a literature search on PubMed using the following (MeSH) terms: personnel selection, general practice, vocational guidance. We focused on findings describing facilitating and inhibitory elements concerning recruitment to general practice.

The findings were used to discuss and adjust the topics in the questionnaire. To broaden diversity in answers, we included two items with free text response options to collect qualitative data.

A draft version of the questionnaire was field-tested in single face-to-face interviews with five junior doctors and four educational experts using a think-aloud test to explore understandability, functionality, and ease of completion [23]. Items perceived as ambiguous were corrected, and additional response options were added to the final questionnaire [23].

### Online questionnaire survey

The final version was converted into an online web-based survey tool encompassing 13 items, in addition to sociodemographics questions. The questionnaire can be found in the Supplemental Material available online.

### Selection of study subjects

The entire population of junior doctors in training positions for general practice in Denmark was invited on 1 July 2015 to participate in the survey. Junior doctors were identified via the official postgraduate medical education register. They received an email invitation with a link to the questionnaire. A reminder was sent after two weeks.

We obtained an anonymized data list with gender, age and graduation university of the total population, allowing us to perform non-responder analysis.

### Coding of qualitative data from open-ended questions

The content of the responses to the open-ended questions was coded thematically according to systematic text condensation using the same strategy as described for the focus group data analysis [21]. Three researchers condensed the identified categories into themes. We compared the degree of coherence between and within the first and second half of the responses to assess data saturation [20,22].

Throughout the coding process, we registered the numbers of statements delivering data to each of the identified themes to be able to estimate the perceived importance of each of the themes.

### Outcomes and statistical analysis

We used descriptive statistics to calculate the distribution of the study population. *T*-test, chi-squared and Fisher’s tests were used to compare responders with non-responders. The responses to the items with fixed response categories were analysed using Friedman’s ANOVA test, *t*-test and chi-squared test. STATA 14 and SPSS version 22.0 were used for the analyses.

## Results

### Results of the questionnaire survey

Of the 1099 invited junior doctors, 670 (61%) completed the questionnaire. The main characteristics of the responders were a mean age of 35.4 years (SD 4.1) and a gender ratio of 3:1 (female/male). There were no statistically significant differences in age and gender distribution between responders and non-responders.

Responders graduating from different universities had significantly different response rates: 55.6–69.3% from Danish universities and 41.9% from universities outside Denmark, *P* = 0.001.

**Figure 2.**
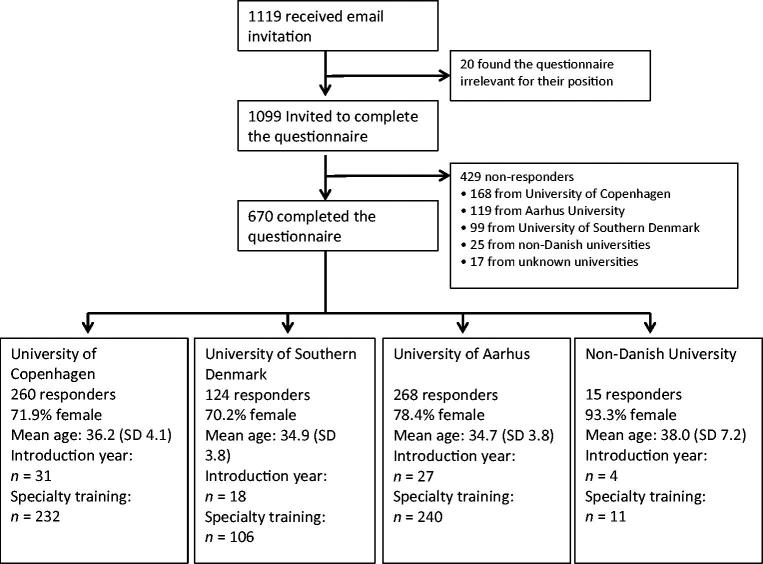
Flow diagram demonstrating, recruitment and the distribution of responders’ vs non-responders’ university of graduation.

The numbers and percentages of responders and non-responders are shown in Figure 2.

### Qualitative results of the questionnaire

We obtained 993 statements in the open-ended questions from 636 out of 670 (94.9%) responders. We detected a massive overlap in statements to the two open-ended questions. The obtained statements are, therefore, reported together. The statements were categorized into seven categories, the number of statements within each of the categories was listed and the seven categories were merged into four themes (Box 2).

Box 2 Categories and emergent themes from the qualitative data in the questionaire survey.

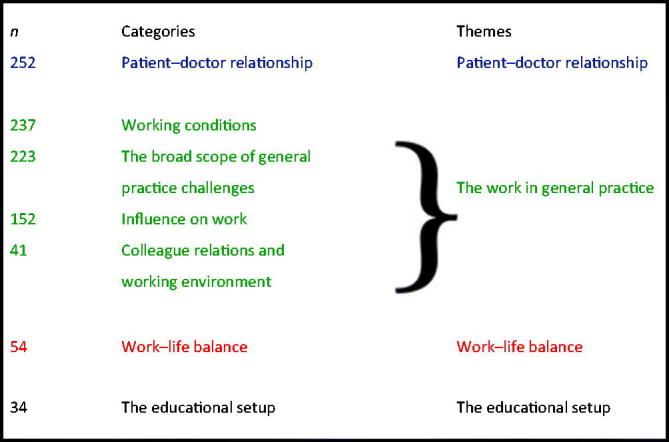
The two open-ended questions providing the statements were:
What do you find to be the most important difference(s) between working in general practice and working at a hospital department? What are the primary reasons for you to choose general practice as your future specialty?
The answers were categorized and emerged into themes as shown. The numbers of statements providing data to each category are shown in the first column to illustrate the perceived importance.

### The patient–doctor relationship

The respondents appreciated the patient–doctor relationship experienced in general practice. They described a unique relationship based on trust and a long-lasting continuity. They experienced that they made a positive difference to their patients.

The consultation room in general practice is exceptional. I am again and again fascinated and humbled by the trust patients’ show in me…. I can follow the course of an illness.

### The work in general practice

The respondents valued the close relationship with colleagues and the long-lasting teamwork at the small, interdisciplinary workplaces where colleagues show personal interest and help each other in a safe, non-competitive environment. They treasured independency, autonomy, and flexibility of a smaller organization where decisions and changes could be made easily and rapidly. Their influence on how to organize their work made them more productive and they valued shouldering leadership for the staff and the patient treatment. They experienced high job satisfaction and well-being.

I thrive on a close relationship with colleagues. The tone and the atmosphere in general practice is more relevant and pragmatic and has room for caring for both colleagues and staff.

Each day, I can improve my clinical practice without having to fight heavy bureaucracy.

The respondents valued the diversity, the range of problems and the exposure to a variety of patients. They stated they found it stimulating to maintain a broad and comprehensive medical knowledge base. They also valued working both on health advocacy and on treatment of diseases.

I love the diversity; patients of all ages and all types of acute and chronic diseases; here I feel I am a real doctor.

### Work–life balance

The respondents stated that both the specialist training programme and the subsequent work in general practice was beneficial for a good work–life balance. They mentioned examples such as work hours compatible with having children and a busy spouse; short commute, few, or no, night shifts.

### The educational setup

The respondents found the GP specialist training programme attractive and preparing them well for general practice providing a broad and comprehensive set of competencies and leading to several career options. The programme is offered locally with no need to commute to university cities.

The comprehensive specialist education provides opportunities to become a private practitioner, to be employed in general practice or a hospital, or combinations of these. It gives me the greatest possible freedom.

Some respondents stated that undergraduate education in general practice was suboptimal. Early exposure to general practice in the mandatory postgraduate basic training had awakened interest in the specialty.

*Quantitative results of the questionnaire.* The views of undergraduate training in general practice received at the different Danish universities are listed in Table 1. In total, 80.4% of junior doctors found that general practice had too little impact on medical curricula and teaching.

**Table 1.** Views on the training in general practice received at the different Danish universities^a^.

Questions	Far too little	Too little	Appropriate	Too much	Far too much
1. Study impact	31.5% (206/655)	47.3% (310/655)	20.6% (136/655)	0.3% (2/655)	0.2% (1/655)
	Not relevant	Less relevant	Relevant	Very relevant	
2. Relevance	3.7% (24/655)	36% (236/655)	52.1% (340/655)	8.2% (55/655)	

Do the responders perceive the following conditions, extracted from the qualitative data in the prior focus-group interviews, as primarily advantageous or disadvantageous?

1. In your opinion, what is the impact of general practice, including its patients and problems, in the medical training at the university where you studied?

2. How relevant did you find the training in general practice compared to working in general practice?

^a^Responders (655) from Danish universities. Responders from universities outside Denmark were excluded.

Junior doctors (75.5%) appreciated that general practice means working in smaller, manageable units, 54.9% preferred being independent and self-employed (Table 2).

**Table 2.** Perception of central characteristics of overall working conditions in general practice

Questions	Primarily an advantage	Equal	Primarily a disadvantage
1. Small working place	75.5% (506/670)	22.4% (150/670)	2.1% (14/670)
2. Independence	54.9% (368/670)	37.5% (252/670)	7.5% (50/670)

Do responders perceive the following conditions, extracted from the qualitative data in the prior focus-group interviews, as primarily advantageous or disadvantageous?

1. General practice is typically a minor organization compared to a hospital department. How do you perceive this in relation to your choice of specialty?

2. As a GP, you are self-employed and responsible for management, organization of work, finance and yourself. How do you perceive this to your choice of specialty?

A total of 82.0% agreed or strongly agreed that they had chosen the specialty to settle as a GP in Denmark (Table 3). Table 3 shows that 93.9% agreed or strongly agreed that it is essential to work with a holistic and patient-centred approach. A total of 64.5% agreed or strongly agreed that basic postgraduate training in general practice had a significant impact on the choice of general practice as a specialty (Table 3). Junior doctors (90.8%) stated that the set-up in the Danish specialist-training programme positively influenced their choice of general practice (Table 3).

**Table 3.** Impact on choice of specialty of different main elements

Statements Do you agree or disagree with the following statements?	Strongly agree	Agree	Neither agree or disagree	Disagree	Strongly disagree
1. Training programme	60.7% (407/670)	30.1% (202/670)	9.1% (61/670)	0.0% (0/670)	0.0% (0/670)
2. Patient-centred approach	69.4% (465/670)	24.5% (164/670)	6.1% (41/670)	0.0% (0/670)	0.0% (0/670)
3. Work as independent GP	51.6% (346/670)	30.4% (204/670)	1.9% (120/670)	0.0% (0/670)	0.0% (0/670)
4. GP in basic training^a^	26.9% (167/620)	37.6% (233/620)	21.5% (133/620)	8.2% (51/620)	5.8% (36/620)

To what extent do the responders agree with the following statements extracted from the qualitative data in the prior focus-group interviews?

1. The quality of the postgraduate training programme in general practice influenced my choice of general practice as a specialty.

2. It is important that I, as a GP, work with a holistic and patient centred approach.

3. I have chosen the specialty because I intend to establish myself as GP in Denmark.

4. Early exposure to general practice in my postgraduate basic training had a significant impact on my decision to choose general practice as a specialty.

a 50 trainees had no basic training in general practice.

GP, general practitioner.

## Discussion

### Main findings

Most of the junior doctors reported that general practice had too little emphasis in the undergraduate curricula. The majority of junior doctors stated that their perception of the comprehensiveness of the GP specialist-training programme influenced the choice of specialty positively as did experiences from early basic postgraduate training in general practice. The junior doctors in general practice valued patient-centred care and a doctor–patient relationship based on continuity and trust. They prioritized working environments, which made the work–life balance feasible. A more substantial part of junior doctors appreciated working in smaller manageable units. Several junior doctors appreciated autonomy and the possibility of organizing and prioritizing their work.

### Strengths and limitations

We find a response rate of 61%, with no statistically significant differences in characteristics to age and gender distribution between respondents and non-respondents, to be acceptable. The significantly lower response rate for doctors who graduated from universities outside Denmark represents only a few persons in the entire population.

We collected both quantitative and qualitative data to obtain a comprehensive understanding. Furthermore, we used a study design ensuring data analysis consensus between several researchers to strengthen the validity of the results.

We had uneven gender participation in the initial focus group interviews 6:1 (female:male) compared to 3:1 in the junior doctor population, but the open-ended questions included in the questionnaire should be able to compensate for possible gender bias from the focus groups.

Caution should always be taken in the interpretation of expressed opinions in questionnaires. However, we combined the quantitative rating of the statements with the open-ended answers and only considered data supported by both approaches as valid.

We only explored the views of the junior doctors once and did not investigate whether the individuals held similar opinions throughout the training programme. Nor did we examine the views of junior doctors who did not choose general practice. These conditions limit our conclusions especially to reasons not to choose general practice.

Our study was conducted in 2015, submitted in 2018 and published in 2019. We think, however, our results are still informative since no major changes have been implemented in general practice or the undergraduate or specialist education since our data collection.

### Findings in relation to other studies

The junior doctors’ preference for a more general practice-oriented undergraduate curriculum is supported by other studies, which argue that a general practice-oriented undergraduate curriculum could have a positive impact on recruitment to general practice [5,8,9,12,24,25]. Recently, a UK study has demonstrated a positive correlation between quantity of GP teaching at medical schools and the percentage of graduates who enter general practice training [9]. Another study has revealed that general practice is given a negative branding in some British medical schools, which may hinder recruitment to general practice [26].

The importance of the postgraduate education set up is supported by a study by Cleland et al. who showed that the right learning environment is highly prioritized by doctors looking for their future specialty [14]. British and Danish studies have also argued that early exposure to general practice in postgraduate training not only enhances recruitment to general practice, it also provides valuable learning for doctors aiming towards other specialities [6,11,12].

We found that future Danish GPs appreciate a patient-centred approach. This is reassuring since previous research has pointed out that a patient-centred approach is the cornerstone in general practice and an excellent platform for high-quality primary healthcare [27]. German and British studies also highlight relation-based, patient-centred care as a positive recruitment parameter [5,26].

The importance of proper working conditions is highlighted in other studies [15,28]. Therefore, it is a potential threat from a recruitment perspective that general practice is experiencing a growing workload, more administrative burdens, and professional fatigue [2].

### Implications

Our findings, and the presented literature, indicate that if more young doctors are to be recruited to general practice, it is relevant to: strengthen the focus of general practice in the undergraduate curriculum; continue development of high-quality educational programmes; consider basic postgraduate training in general practice for all newly graduated doctors; safeguard the doctor–patient relationship based on continuity; safeguard the patient-centred approach; and ensure good working conditions including the possibility for the GPs to organize and prioritize their work.

Future studies should explore whether the amount and quality of general practice training in universities can enhance recruitment to general practice. We need to know more about the views of doctors who choose other specialities. Also, the impact of other recruitment initiatives should be explored.

## Conclusion

The following factors seem to influence the choice of general practice: a doctor–patient relationship based on continuity and patient-centred primary healthcare, good educational environments and a feasible work–life balance and the possibility of organizing their work in smaller manageable units.

Plausibly, increased undergraduate study time and clinical work in general practice together with basic postgraduate training in general practice and a comprehensive specialist-training programme could have a positive influence on the choice of general practice as a speciality.

## Supplementary Material

Questionnaire (English translation) used for online survey
