# Supplementary material for: Why do Danish junior doctors choose general practice as their future specialty? Results of a mixed-methods survey
Source: Eur J Gen Pract. 2019 Jul 24;25(3):149–56. doi: 10.1080/13814788.2019.1639668 (PMC6713188; doi:10.1080/13814788.2019.1639668)
Supplement: Questionnaire (English translation) used for online survey [file IGEN_A_1639668_SM0086.docx]

**Appendix**

**The questionnaire (English translation) used for online survey**

1.

When did you first interest in general practice as a specialty start? (only one answer)

First part of undergraduate study:

Second part of undergraduate study:

First part of internship:

Second part of internship:

Later:

2.

On a scale from 0 to 100 how sure were you, that you would apply for a GP specialist training position at the following times?

At the end of the last undergraduate

0-100

At the end of the first part of internship

0-100

At the end of the first part of internship

0-100

At the end of the GP introduction training position

0-100

3.

If you have had an introduction training position in another specialty after your GP introduction training how sure were you at the end of this introduction position that you would apply for GP specialty training? (only one answer)

0-100

or

I have not had another introduction training position after my GP introduction training (mark)

4.

In your opinion, what is the impact of general practice, including its patients and problems, in the medical training at the university where you studied? (only one answer)

Way too little

Too little

Appropriate

Too much

Way too much

5.

How relevant did you find training in general practice compared to working in general practice? (only one answer)

Not relevant

Less relevant

Relevant

Very relevant

6.

What do you find to be the most important difference(s) between working in general practice and working at a hospital department?
(open-ended text)

7.

What are the primary reasons for you to choose general practice as your future specialty?

(open-ended text)

8.

A general practice is typically a minor organisation compared to a hospital department. How do you perceive this in relation to your choice of specialty? (only one answer)

Primarily an advantage

Equal

Primarily a disadvantage

9.

As a GP, you are self-employed and responsible for management, organisation of work, finance and for yourself. How do you perceive this in relation to your choice of specialty? (only one answer)

Primarily an advantage

Equal

Primarily a disadvantage

To what extent do you agree with the following statements?

10.

The quality of the postgraduate training programme in general practice influenced my choice of general practice as specialty. (only one answer)

Strongly agree

Agree

Neither agree or disagree

Disagree

Strongly disagree

11.

It´s important that I, as a GP, work with a holistic and patient centred approach. (only one answer)

Strongly agree

Agree

Neither agree or disagree

Disagree

Strongly disagree

12.

I have chosen the specialty because it´s my intention to establish myself as GP in Denmark. (only one answer)

Strongly agree

Agree

Neither agree or disagree

Disagree

Strongly disagree

13.

Early exposure to general practice in my postgraduate basic training had a significant impact on my decision to choose general practice for my career. (only one answer)

Strongly agree

Agree

Neither agree or disagree

Disagree

Strongly disagree

Not able to answer since I have not been in general practice as a part of my postgraduate basic training

*Background data*

Year of graduation (number)

Graduated from which university

(University of Aarhus, University of Copenhagen, University of Southern Denmark or Foreign University)

Age (number)

Gender (female or male)

In which region in Denmark do you work (Capital Region of Denmark, Region Zealand, Southern Region of Denmark, Central Denmark Region or North Denmark Region)

Actual position (introduction training or specialty training)

General practice was a part of min internship (yes or no)
